# Quantitative MRI for Monitoring Metabolic Dysfunction‐Associated Steatotic Liver Disease: A Test–Retest Repeatability Study

**DOI:** 10.1002/jmri.29610

**Published:** 2024-09-25

**Authors:** Cayden Beyer, Anneli Andersson, Elizabeth Shumbayawonda, Naim Alkhouri, Ami Banerjee, Prashant Pandya, Mukesh Harisinghani, Kathleen Corey, Andrea Dennis, Michele Pansini

**Affiliations:** ^1^ Perspectum Ltd Oxford UK; ^2^ Arizona Liver Health Chandler Arizona USA; ^3^ University College London Hospitals NHS Trust London UK; ^4^ Institute of Health Informatics University College London London UK; ^5^ Barts Health NHS Trust, The Royal London Hospital London UK; ^6^ Massachusetts General Hospital Boston Massachusetts USA; ^7^ Clinica Di Radiologia EOC, Istituto Di Imaging Della Svizzera Italiana (IIMSI), Ente Ospedaliero Cantonale Lugano Switzerland; ^8^ John Radcliffe Hospital, Oxford University Hospitals NHS Foundation Trust Oxford UK

**Keywords:** cT1, PDFF, multiparametric‐MRI, MASH, MASLD

## Abstract

**Background:**

Quantitative magnetic resonance imaging metrics iron‐corrected T1 (cT1) and liver fat from proton density fat‐fraction (PDFF) are both commonly used as noninvasive biomarkers for metabolic dysfunction‐associated steatohepatitis (MASH); however, their repeatability in this population has rarely been characterized.

**Purpose:**

To quantify the variability of cT1 and liver fat fraction from PDFF in patients with biopsy‐confirmed metabolic dysfunction‐associated steatotic liver disease (MASLD) and MASH.

**Study Type:**

Prospective, single center.

**Population:**

Twenty‐one participants (female = 11, mean age 53 ± 24 years) with biopsy‐confirmed MASLD, including 6 with MASH and fibrosis ≥2.

**Field Strength/Sequence:**

3 T; T1 and T2* mapping for the generation of cT1 (shMOLLI: CardioMaps and 2D MDE, T1map‐FIESTA and LMS MOST: StarMap, 2D Multi‐Echo FSPGR) and magnitude‐only PDFF sequence for liver fat quantification (LMS IDEAL: StarMap, 2D Multi‐Echo FSPGR).

**Assessment:**

T1 mapping and PDFF scans were performed twice on the same day for all participants (*N* = 21), with an additional scan 2–4 weeks later for MASH patients with fibrosis ≥2 (*N* = 6). Whole liver segmentation masks were generated semi‐automatically and average pixel counts within these masks were used for the calculation of cT1 and liver fat fraction.

**Statistical Tests:**

Bland–Altman analysis for repeatability coefficient (RC) and 95% limits of agreement (LOA) and intraclass correlation coefficient (ICC).

**Results:**

Same‐day RC was 32.1 msec (95% LOA: −36.6 to 24.2 msec) for cT1 and 0.6% (95% LOA: −0.5% to 0.7%) for liver fat fraction; the ICCs were 0.98 (0.96–0.99) and 1.0, respectively. Short‐term RC was 65.2 msec (95% LOA: −63.8 to 76.5 msec) for cT1 and 2.6% (95% LOA: −2.8% to 3.1%) for liver fat fraction.

**Data Conclusion:**

In participants with MASLD and MASH, cT1 and liver fat fraction measurements show excellent test–retest repeatability, supporting their use in monitoring MASLD and MASH.

**Level of Evidence:**

2

**Technical Efficacy:**

Stage 2

## Graphical abstract

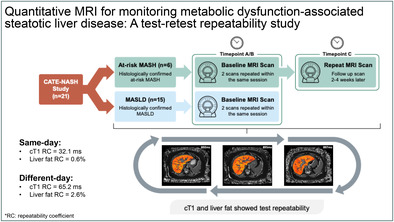

Metabolic dysfunction‐associated steatotic liver disease (MASLD) is reaching epidemic levels, affecting an estimated one billion people worldwide,^1^, ^2^ with the more aggressive form, metabolic dysfunction‐associated steatohepatitis (MASH), affecting 3%–12% of Americans.^3^ MASH can lead to dangerous outcomes, including decompensated cirrhosis,^4^, ^5^ increased risk of heart disease,^6^ and liver cancer. This year, the United States Food and Drug Administration (FDA) approved oral resmetirom as the first treatment for MASH with moderate‐to‐advanced fibrosis,^7^ adding a single pharmacological tool to the limited arsenal against steatotic liver disease. Diagnosis and staging is currently dependent on invasive liver biopsy, which is neither acceptable to patients nor scalable. To bring new treatments safely and effectively to patients, noninvasive diagnostic and monitoring tools are urgently needed.^8^

Quantitative magnetic resonance imaging (MRI) has been shown as a safe alternative to liver biopsy for liver tissue characterization. T1 mapping measures longitudinal relaxation time for protons after excitation by a radiofrequency pulse.^9^ Increases in the native T1 relaxation time may be due to increases in water content in biological tissues, which can be indicative of disease pathology such as oedema, inflammation, and/or fibrosis.^10^, ^11^ cT1 builds on standard T1 mapping, with standardization algorithms to account for the confounder, iron, and to standardize the measure across field strength and manufacturer. cT1 has been referred to as a marker of fibro‐inflammation owing to correlations with all the histopathologic features of MASLD^11^; thus, it is a good diagnostic biomarker, with values greater than 800 msec indicating a high chance of MASLD, and over 875 msec associated with an increased risk of fibrotic MASH.^11^ A change in cT1 of ≥80 msec has been associated with meaningful histological changes.^12^, ^13^ MRI proton density fat fraction (PDFF) is the ratio of MR visible protons associated with fat to all MR visible protons and is expressed as a percentage. Liver fat from PDFF correlates very well with histological grading of steatosis levels,^14^ with a threshold of ≥5% indicating hepatic steatosis,^15^ and ≥8% is commonly used as an indicator of MASH.^16^ Change in liver fat using PDFF is commonly reported as the relative reduction from baseline with a drop of ≥30% associated with increased odds of meeting a meaningful change based on histological endpoints.^17^

Several trials have already employed cT1 and liver fat from PDFF as secondary or exploratory endpoints in MASH.^18^, ^19^, ^20^ Clinically meaningful reductions in cT1 have been observed in response to interventions including bariatric surgery,^21^ low energy diets,^22^ and in experimental compounds targeting liver‐specific fat reduction^23^, ^24^ and antifibrotic therapies,^12^, ^25^ with changes observed as early as 8 weeks.^25^ Similarly, large reductions in liver fat with treatment have been observed in many clinical trials, especially those with a metabolic mechanism of action, such as FGF‐21 analogues,^26^ THR‐beta receptor agonists,^7^ and GLP‐1s.^27^ Liver fat is also modifiable with lifestyle with evidence of significant reductions in patients with steatotic liver disease following a Mediterranean diet.^28^

While in clinical trials, responder analysis is often used to establish a clinically meaningful change, in clinical practice, it is also important to know the smallest detectable difference. While both biomarkers have already been established to have high linearity and test–retest repeatability in healthy cohorts,^29^ data on the measurement error expected in patients with MASH are not well characterized. This information will be pertinent if these tools are to be used in monitoring response to treatments as new therapies become available. This study aimed to quantify the variability of biomarkers in patients with biopsy‐confirmed MASLD and MASH to provide an interpretation of the amount of change that represents a true change in the health of the liver, thereby offering a guideline for its interpretation in clinical trials and everyday clinical practice.

## Materials and Methods

### Study Design and Participants

**Figure 1:**
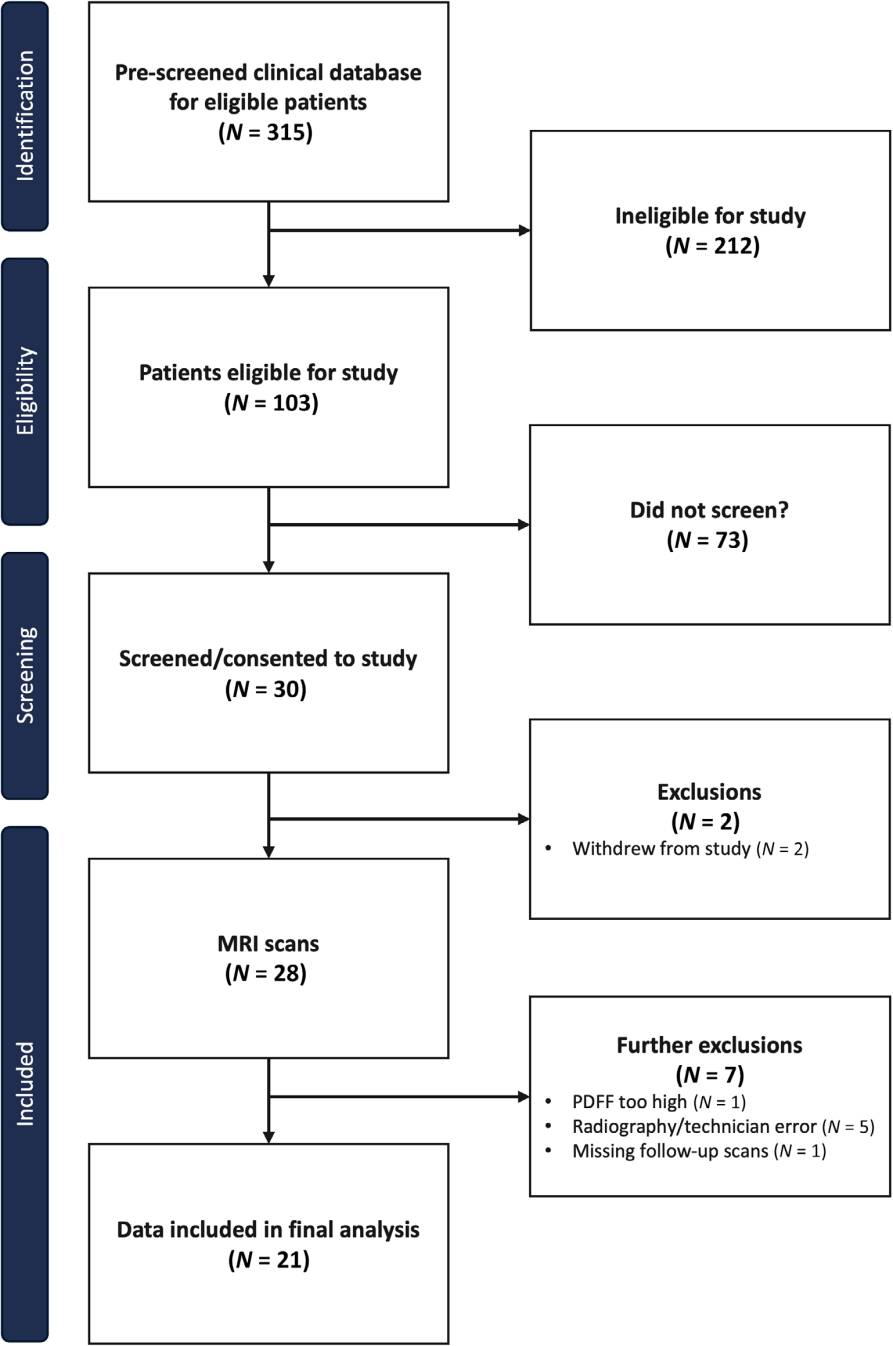
Consort diagram of subject omissions due to missing data for all phases of the study.

This prospective, observational, single‐site study (CATE‐NASH; NCT04341246) was funded by the FDA as part of the biomarker qualification program. Inclusion criteria included patients with biopsy‐confirmed MASLD (evidence of steatosis in biopsy determined within 6 weeks of scanning) below the age of 75 years and with no contraindications to MRI. Following enrolment and liver biopsy, all participants underwent multiparametric MRI for liver tissue characterization (Fig. 1). All MRI scans were performed on the same scanner. All participants were scanned twice on the same day (at least 5 minutes apart but remaining on the scanner), and those with a biopsy‐confirmed diagnosis of MASH (evidence of steatosis, lobular inflammation, and hepatocellular ballooning on biopsy, NAFLD activity score ≥4) with fibrosis ≥2 were invited to a follow‐up scan 2–4 weeks later. There were no other interventions in addition to standard clinical care. Patients were not permitted to be enrolled in a clinical trial during the study. All clinical investigations were conducted in accordance with the Declaration of Helsinki 2013 and approved by the relevant local institutional review boards. Written informed consent was obtained from all participants.

### Image Acquisition and MRI Protocol

All participants underwent abdominal multiparametric MRI using a 3 T scanner (Signa Premier, GE Healthcare, Waukesha, WI, USA). The protocol included sequences for calculating iron‐corrected T1 (cT1) and liver fat fraction. The cT1 calculation used shMOLLI (CardioMaps and 2D MDE, T1map‐FIESTA) and LiverMultiScan (LMS) MOST (StarMap, 2D Multi‐Echo FSPGR) sequences. Liver fat content was measured using magnitude‐only proton density fat fraction (MAGO‐PDFF) with the LMS IDEAL sequence (StarMap, 2D Multi‐Echo FSPGR); full acquisition details are in the Supplemental Material. All MR scans were performed with participants lying supine following a 4‐hour fast. The average scan time was 10 minutes. The specific MRI protocol used has been described elsewhere.^29^ cT1 and fat fraction maps were generated using commercially available software (LiverMultiScan, Perspectum). To enable a median value across the liver to be calculated, generated cT1 maps were delineated into whole liver segmentation maps using a semi‐automatic, deep learning method (U‐net), with nonparenchyma structures such as bile ducts and large blood vessels as well as image artifacts excluded. The pixel values from these segmentation maps were averaged, and the median values were reported.

### Statistical Analyses

Analyses were performed using R software version 4.3.1 (R Foundation for Statistical Computing, Vienna, Austria). Mean and standard deviation (SD) were used to describe normally distributed continuous variables, median and interquartile range were used to describe non‐normally distributed continuous variables, and frequency and percentages were used for categorical variables. Continuous variables compared across subgroups using Wilcoxon rank sum test and categorical variables compared using Fisher's exact test. cT1 and PDFF both follow a Gaussian distribution. The same day and between‐day repeatability of cT1 and liver fat measurements were assessed. The same day and between‐day differences of cT1 and liver fat also follow a normal distribution. The repeatability coefficient (RC), bias, 95% limits of agreement (LOA), and within‐subject SD (wSD) were derived from the Bland–Altman method, intraclass correlation coefficient (ICC), and coefficient of variation (*r* ^2^) were calculated to quantify the repeatability of both metrics. LOA were reported as the mean difference between the two timepoints ± 1.96 × SD of the differences. RCs were calculated following the methods described elsewhere,^30^ the results are reported both in absolute values and as relative changes. Interclass correlation coefficients (ICCs) and 95% confidence intervals are also reported for all comparisons. ICC coefficients were calculated utilizing a two‐way mixed effects model and are similarly presented as averages across the repeatability experiments. As a guide, ICC values of less than 0.50, between 0.50 and 0.75, between 0.75 and 0.90, and greater than 0.90 are indicative of poor, moderate, good, and excellent reliability, respectively.^31^ The diagnostic accuracy for identifying patients with MASH with fibrosis ≥2 was also assessed for each biomarker using the area under the receiver operating characteristic curve (AUROC); sensitivity and specificity of the recommended thresholds for identifying patients in clinical trials (cT1 > 875 msec^11^ and liver fat fraction ≥8%^16^) were reported. The estimated sample size required for Bland–Altman estimates on variability was 10 participants, this was based on the methodology from Lu et al^32^ and based on worst case cT1 reproducibility in literature^29^ (assuming power = 0.8, mean of differences = 15.44, SD of differences = 52.64, alpha level of Bland–Altman LOA 0.05, alpha level of LOA confidence interval 0.99).

## Results

### Cohort Description

**Figure 2:**
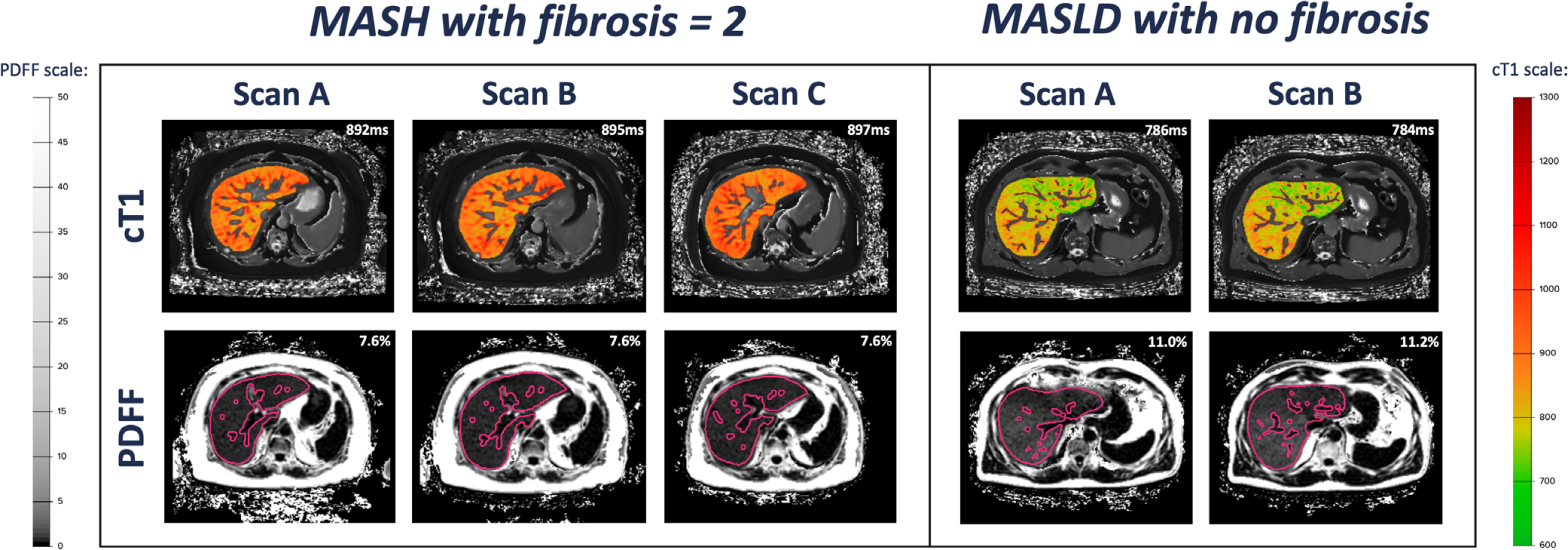
Example cT1 and PDFF maps of a patient with MASLD and no fibrosis and an example patient with confirmed MASH and fibrosis of 2.

Twenty‐one participants (female = 11, mean age 53 ± 24 years) with MASLD were included in the full analysis. The mean body mass index 36.8 kg/m^2^, and 43% were diabetic. Six out of 21 participants had MASH with fibrosis ≥2 based on their local biopsy and received a third MRI scan 3 weeks later on average (SD: 2–4). A complete description of the cohort characteristics is provided in Table 1 and a breakdown of histology scores in Table S1 in the Supplemental Material. Representative MRI images of all participants are shown in Fig. 2.

**Table 1:** Participants' Demographics and Baseline Characteristics

	Full Cohort (*N* = 21)	MASH With Fibrosis ≥2 (*N* = 6)	MASLD/MASH With Fibrosis <2 (*N* = 15)	*P*‐Value
Age (years)	53 (24)	43 (35)	54 (12)	0.76
Female, *N* (%)	11 (52%)	4 (67%)	7 (47%)	0.64
Race				
White	18 (90%)	4 (80%)	14 (93%)	0.45
Hawaiian/Pacific Islander	1 (5.0%)	0 (0%)	1 (6.7%)	
NA	1 (5.0%)	1 (20%)	0 (0%)	
Ethnicity				
White of any ethnic region	16 (76%)	3 (50%)	13 (87%)	0.053
Hispanic/Latino	4 (19%)	3 (50%)	1 (6.7%)	
Any other ethnic group	1 (4.8%)	0 (0%)	1 (6.7%)	
BMI (kg/m^2^)	36 (7)	38 (9)	35 (7)	0.47
Waist circumference (cm)	110.4 (12.8)	110.3 (17.3)	110.4 (11.3)	0.67
Type‐2 diabetes, *N* (%)	9 (43%)	3 (50%)	6 (40%)	0.99
Hypertension, *N* (%)	11 (52%)	4 (67%)	7 (47%)	0.64
Dyslipidemia, *N* (%)	12 (57%)	4 (67%)	8 (53%)	0.66
ALT (U/L)	48 (110)	105 (122)	37 (44)	0.17
AST (U/L)	34 (38)	66 (55)	29 (14)	0.053
MRI‐derived metrics				
cT1 (msec)	861 (87)	901 (47)	846 (95)	0.15
LFF (%)	11.0 (5.6)	11.9 (4.0)	11.0 (7.5)	0.68
T2* (msec)	21 (3)	22 (4)	21 (3)	0.51

*P*‐value <0.05 indicate statistical difference between MASLD/MASH with fibrosis <2 and MASH with fibrosis ≥2 groups.

BMI = body mass index; cT1 = iron‐corrected T1 mapping; IQR = interquartile range; LFF = liver fat fraction; MASH = metabolic dysfunction‐associated steatohepatitis; MASLD = metabolic dysfunction‐associated steatotic liver disease; NAS = nonalcoholic steatohepatitis activity score; SD = standard deviation.

### Diagnostic Accuracy of cT1 and Liver Fat Fraction to Identify MASH With Fibrosis

Mean cT1 was 861 ± 87 msec and liver fat fraction was 11 ± 5.6%. The cT1 AUROC for separating those with MASLD/MASH and fibrosis <2 from MASH with fibrosis ≥2 was 0.71, and the recommended threshold of cT1 > 875 msec had a sensitivity of 0.83 and a specificity of 0.60. The AUROC for liver fat fraction was 0.57, and the 8% cutoff point had a sensitivity of 0.83 and specificity of 0.27.

### Same‐Day Test Retest Repeatability

**Figure 3:**
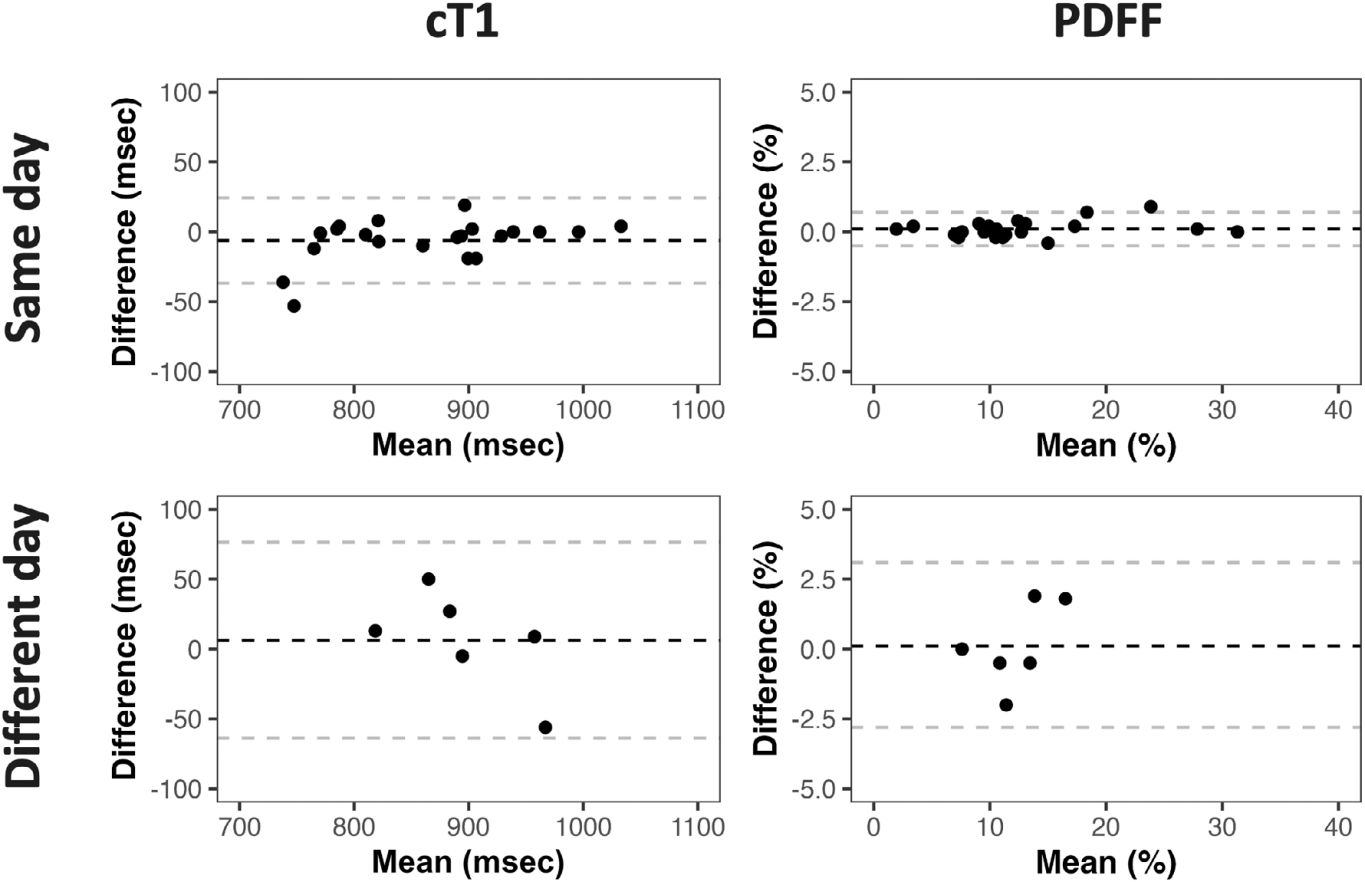
Bland‐altman plots showing cT1 and PDFF difference between baseline and follow‐up on the same day (N=21), top row; Baseline and follow‐up on a different day (N=6), bottom row. The dashed lines represent the upper and lower 95% limits of agreement and the mean difference.

Same‐day scans were performed, with an average time of 5 minutes between scans. Both cT1 and liver fat fraction demonstrated high test retest repeatability and low bias (cT1: wCV: 1.5%; RC: 32.1 msec [95% LOA: −36.6 to 24.2 msec]; liver fat fraction: wCV: 1.9%; RC: 0.6% [95% LOA: −0.5% to 0.7%], expressed in relative terms: RC: 5.3%). The full statistics are presented in Table 2 and Fig. 3.

**Table 2:** Repeatability Statistics of cT1 and Liver Fat From PDFF for Same Day and Different Day Scanning

	Bias	wSD	RC (95% LOA)	wCV	ICC (95% LOA)	*r* ^2^
cT1 (msec)						
Same day (*N* = 21)	−6.2 msec	11.6 msec	32.1 msec (−36.6, −24.2)	1.5%	0.98 (0.96, 0.99)	0.97
Different day (*N* = 6)	6.3 msec	23.5 msec	65.2 msec (−63.8, −76.5)	2.6%		0.77
LFF (%)						
Same day (*N* = 21)	0.1%	0.2%	0.6% (−0.5, − 0.7)	1.9%	1.00 (1.00, 1.00)	1.00
Different day (*N* = 6)	0.1%	0.9%	2.6% (−2.8, − 3.1)	7.4%		0.84

cT1 = iron‐corrected T1 mapping; ICC = intraclass correlation coefficient; LFF = liver fat fraction; LOA = limits of agreement; PDFF = proton density fat‐fraction; *r* ^2^ = coefficient of determination; RC = repeatability coefficient; wSD = within‐subject standard deviation; wCV = within‐subject coefficient of variation.

### Between‐Day Test Retest Repeatability

Between‐day scans were performed at an average of 3 weeks apart and on six participants with MASH and fibrosis stage 2. cT1 demonstrated high between‐day repeatability (wCV: 2.6%; RC: 65.2 msec [95% LOA: −63.8 to 76.5 msec]; Table 2 and Fig. 3) and liver fat fraction and good between‐day repeatability (wCV: 7.4%; RC: 2.6% [95% LOA: −2.8% to 3.1%], expressed in relative terms: RC: 20.5%).

## Discussion

This study aimed to report the measurement repeatability of two commonly used imaging biomarkers in MASH, cT1, and liver fat fraction from PDFF, in patients with MASLD. This data extends on existing knowledge as it is the first‐time repeated measures were performed on biopsy‐confirmed MASLD patients. The results showed that both biomarkers exhibit very low variability in repeated measurements on the same scanner.

Specifically, the cT1 same‐day RC was 32 msec (−36.6, 24.2), and liver fat fraction from PDFF was 0.6% (−0.5, 0.7). Both are much lower than previously reported values in healthy participants (cT1: 46 msec^29^; PDFF: 1.2% from magnitude only PDFF^33^ 0.8%^29^), although this is perhaps to be expected given that data from only one MRI scanner was acquired in this study. The interpretation of the RC in this context is the clinical cutoff point used to discern between changes in a biomarker's measurements due to measurement error versus changes that exceed measurement error, thus indicating real change in the patient.^34^ As such these values have direct application in the clinical management of patients undergoing therapy and in drug development to inform individual responder analysis. These are not to be confused with clinically meaningful changes, which is the magnitude of change that best predicts an outcome of clinical relevance, such as histological changes that are currently used in MASH.

In this study, we also attempted to characterize the expected variability in a measurement when introducing some day‐to‐day biological noise by inviting patients with MASH with fibrosis back for a scan a few weeks later. This is perhaps more important in clinical practice and drug development, particularly in MASH, where clinical trials often suffer from high placebo response rates, deemed to be potentially related to lifestyle changes that people make when they know they are being assessed (Hawthorn effect). Our results showed increased variability, with RCs for cT1 of 62 msec and liver fat fraction of 2.6%. Expressed relative to the average PDFF in this study (11%), this equates to a 22% relative difference between measurements. While these results are only on six participants, these data are underpowered to be conclusive, the magnitude of the relative change that can occur over a few weeks in participants not undergoing intervention may imply that the recommended clinically meaningful relative reduction in liver fat fraction of 30% may perhaps be too low and too easy to achieve with simple changes in behavior. In fact, studies have reported between 21% and 33% of participants in placebo arms of clinical trials achieving a 30% relative reduction in liver fat^7^, ^27^, ^35^ resulting in updated suggestions that a 50% relative reduction in liver fat fraction may be more suitable as an indicator resolution of MASH.^26^ The cT1 repeatability was also greater for the six patients scanned a few weeks apart; however, at 62 msec, this was still considerably under the reported threshold for meaningful change in cT1 of ≥80 msec.^13^ The average between‐day difference (or measurement bias) for cT1 was −6 msec (23.5 msec). This aligns with average changes in cT1 reported in placebo groups, for example. −6.1 msec (±11.7) and −9.9 msec (95% confidence interval: −35.5 to 18.7)^36^, ^37^ have been reported in two recent phase II clinical trials.

This is the first time that the test–retest repeatability of cT1 has been explored in a histologically confirmed MASLD cohort. The results are encouraging and add confidence in the measure as a reliable metric for managing MASH in longitudinal studies. The RCs for both metrics were low for same‐day and between‐day scans, implying that cT1 is not too sensitive to small biological changes introduced through day‐to‐day activities. Such consistency is crucial for clinical applications, particularly when treatments become available for MASH. This study also reported the diagnostic accuracy of both cT1 and liver fat fraction to identify those with MASH with fibrosis of 2 or greater. The results showed cT1 to have a higher AUC and specificity than liver fat fraction. This is likely because liver fat fraction from PDFF has been well reported to have a nonlinear relationship with increasing fibrosis,^13^, ^14^, ^38^ specifically it increases up to F2 and then begins to decline (Fig. 3).

### Limitations

Our study has some limitations. The experiment was only conducted on a single MRI scanner, and the power was small to establish definitive test–retest repeatability on different days for those with MASH and fibrosis ≥2. Moreover, MOLLI T1 mapping sequences demonstrate high sensitivity to changes in hepatic lipid content, especially when liver fat levels are elevated. However, this characteristic does not diminish the utility of the measure in MASLD and it is not known to affect cT1 repeatability.

## Conclusions

In conclusion, our results quantify the minimal detectable (absolute) changes in cT1 (32 msec) and liver fat fraction (0.6%) that discern measurement error from real change when monitoring patients with MASLD on a single scanner. Generally accepted confounds such as natural diurnal or day‐to‐day fluctuations, however, may lead to higher changes. However, in practice, any absolute change above these values represents a real change.

## Author Contributions

Andrea Dennis: conceptualization, formal analysis, methodology, project administration, supervision, writing the original draft, review and editing. Cayden Beyer: data curation, formal analysis, methodology, software development, validation and visualization, writing the original draft, review and editing. Anneli Andersson: methodology. Elizabeth Shumbayawonda: methodology, review and editing. Michele Pansini: methodology, project administration, supervision, writing the original draft, review and editing. Kathleen Corey: project administration, review and editing. Prashant Pandya: project administration, review and editing. Naim Alkhouri: review and editing. Ami Banerjee: review and editing. Mukesh Harisinghani: review and editing.

## Conflict of Interest

Cayden Beyer, Anneli Andersson, Elizabeth Shumbayawonda, and Andrea Dennis are all employees of Perspectum. Naim Alkhouri, Mukesh Harisinghani, and Michele Pansini are consultants for Perspectum.

## Supporting information


**Data S1**. Supporting Information.
