# Supplementary material for: Quantitative MRI for Monitoring Metabolic Dysfunction‐Associated Steatotic Liver Disease: A Test–Retest Repeatability Study
Source: J Magn Reson Imaging. 2024 Sep 25;61(4):1947–55. doi: 10.1002/jmri.29610 (PMC11896917; doi:10.1002/jmri.29610)
Supplement: Supplementary file 1 — Data S1. Supporting Information. [file JMRI-61-1947-s001.docx]

**Quantitative MRI as a tool for monitoring metabolic dysfunction-associated steatotic liver disease: A test-retest repeatability study**

**MATERIALS AND METHODS**

**Image acquisition parameters**

All participants underwent abdominal multiparametric MRI using a 3T scanner (GE Medical Systems Signa Premier). The protocol included sequences for calculating iron-corrected T1 (cT1) and liver fat content. T

The cT1 calculation used shMOLLI (CardioMaps and 2D MDE, T1map-FIESTA) and LMS MOST (StarMap, 2D Multi-Echo FSPGR) sequences.

Liver fat content was measured using magnitude-only proton density fat fraction (MAGO-PDFF) with the LMS IDEAL sequence (StarMap, 2D Multi-Echo FSPGR).

- The shMOLLI sequence used a 128x144 acquisition matrix, with RBW of 83.33 kHz, ASSET of 2, and 8 mm slice thickness. The flip angle was 35°, FOV 44 cm, and Phase FOV 0.75, capturing 5 slices. TE was set to Min Full, with an initial TI of 103 ms and TI increment of 80 ms.
- The LMS MOST sequence employed a 128x128 acquisition matrix, maintaining the same RBW. ARC was set to 2, with a reduced slice thickness of 3 mm. The flip angle was 9°, and Phase FOV increased to 0.80, capturing 12 slices. TE was set to Minimum, with 8 echoes and a TR of 10.7 ms.
- LMS IDEAL utilized a 128x128 acquisition matrix, increased RBW to 125 kHz, and ARC of 2. Slice thickness was 10 mm, with a flip angle of 3°. FOV remained at 44 cm, with Phase FOV at 0.90, capturing 5 slices. TE was set to Min Full, using 12 echoes and a TR of 16.7 ms.

**Supplementary Table 1: Overview of participants histology scores.** P-value < 0.05 indicates statistical difference between MASLD/MASH with fibrosis < 2 and MASH with fibrosis ≥ 2 groups.

MASH: metabolic dysfunction-associated steatohepatitis; MASLD: metabolic dysfunction-associated steatotic liver disease; NAS: non-alcoholic steatohepatitis activity score

|  | **Full Cohort**  **(n = 21)** | **MASH with fibrosis ≥ 2**  **(n = 6)** | **MASLD/MASH with fibrosis < 2**  **(n = 15)** | **P-value** |
| --- | --- | --- | --- | --- |
| **Steatosis Score** |  |  |  | 0.56 |
| 0 (< 5%) | 0 (0%) | 0 (0%) | 0 (0%) |  |
| 1 (5–33%) | 8 (38%) | 2 (33%) | 6 (40%) |  |
| 2 (33–66%) | 11 (52%) | 4 (67%) | 7 (47%) |  |
| 3 (> 66%) | 2 (9.5%) | 0 (0%) | 2 (13%) |  |
| **Inflammation Score** |  |  |  | 0.15 |
| 0 | 5 (24%) | 0 (0%) | 5 (33%) |  |
| 1 | 10 (48%) | 2 (33%) | 8 (53%) |  |
| 2 | 5 (24%) | 3 (50%) | 2 (13%) |  |
| 3 | 1 (4.8%) | 1 (17%) | 0 (0%) |  |
| **Ballooning Score** |  |  |  | 0.003 |
| 0 | 4 (19%) | 0 (0%) | 4 (27%) |  |
| 1 | 12 (57%) | 2 (33%) | 10 (67%) |  |
| 2 | 5 (24%) | 4 (67%) | 1 (6.7%) |  |
| **NAS*** |  |  |  | 0.029 |
| 0 | 0 (0%) | 0 (0%) | 0 (0%) |  |
| 1 | 2 (9.5%) | 0 (0%) | 2 (13%) |  |
| 2 | 1 (4.8%) | 0 (0%) | 1 (6.7%) |  |
| 3 | 6 (29%) | 0 (0%) | 6 (40%) |  |
| 4 | 4 (19%) | 1 (17%) | 3 (20%) |  |
| 5 | 5 (24%) | 3 (50%) | 2 (13%) |  |
| 6 | 3 (14%) | 2 (33%) | 1 (6.7%) |  |
| **Fibrosis Stage†** |  |  |  | 0.05 |
| 0 | 8 (38%) | 0 (0%) | 8 (53%) |  |
| 1 | 7 (33%) | 0 (0%) | 7 (47%) |  |
| 2 | 6 (29%) | 6 (100%) | 0 (0%) |  |

* No participants had NAS 7 or 8. †: No participants had stage 3 or 4 fibrosis.
